# Severe hypercalcemia associated with hypophosphatemia in very premature infants: a case report

**DOI:** 10.1186/s13052-021-01104-6

**Published:** 2021-07-07

**Authors:** Nicola Improda, Francesca Mazzeo, Alessandro Rossi, Claudia Rossi, Francesco Paolo Improda, Angelo Izzo

**Affiliations:** 1 Department of Translational Medical Sciences, Section of Pediatrics, Federico II University of Naples, Naples, Italy; 2 Pediatric Endocrinology Unit, Department of Translational Medical Sciences, University of Naples “Federico II”, Via S. Pansini, 5, 80131 Naples, Italy; 3 Department of Medicine, Surgery and Dentistry “Scuola Medica Salernitana”, University of Salerno, Baronissi, SA Italy; 4 Department of Ophthalmology, Umberto I Hospital, Nocera Inferiore, Italy; 5 Department of Public Health, Federico II University of Naples, Naples, Italy; 6 Neonatal Intensive Care Unit, Malzoni Medical Center, Avellino, Italy

**Keywords:** Hypercalcemia, Extremely low birth weight, Hypophosphatemia, Small for gestational age, Case report

## Abstract

**Background:**

Severe hypercalcemia is rare in newborns; even though often asymptomatic, it may have important sequelae. Hypophosphatemia can occur in infants experiencing intrauterine malnutrition, sepsis and early high-energy parenteral nutrition (PN) and can cause severe hypercalcemia through an unknown mechanism. Monitoring and supplementation of phosphate (PO4) and calcium (Ca) in the first week of life in preterm infants are still debated.

**Case presentation:**

We report on a female baby born at 29 weeks’ gestation with intrauterine growth retardation (IUGR) experiencing sustained severe hypercalcemia (up to 24 mg/dl corrected Ca) due to hypophosphatemia while on phosphorus-free PN. Hypercalcemia did not improve after hyperhydration and furosemide but responded to infusion of PO4. Eventually, the infant experienced symptomatic hypocalcaemia (ionized Ca 3.4 mg/dl), likely exacerbated by contemporary infusion of albumin. Subsequently, a normalization of both parathyroid hormone (PTH) and alkaline phosphatase (ALP) was observed.

**Conclusions:**

Although severe hypercalcemia is extremely rare in neonates, clinicians should be aware of the possible occurrence of this life-threatening condition in infants with or at risk to develop hypophosphatemia. Hypophosphatemic hypercalcemia can only be managed with infusion of PO4, with strict monitoring of Ca and PO4 concentrations.

## Background

Severe hypercalcemia in newborns is a rare event and, even though generally well tolerated, it may be lethal or cause several complications, such as bradycardia, seizures, renal and brain calcifications [1–5]. Therefore, appropriate investigations and treatment strategies should be promptly established.

Extremely (E-) or very (V-) low birth weight (LBW) neonates, especially if born small for gestational age (SGA) are more prone to develop hypophosphatemic hypercalcemia [1], due to a complex combination of factors, such as depleted phosphate (PO4) stores, renal immaturity, increased infection risk and early high-energy parenteral nutrition (PN) [2–4]. However, due to the rarity of the condition, the exact mechanism underlying the association between hypophosphatemia and severe hypercalcemia is still poorly understood.

Optimal monitoring and nutritional strategies to prevent early hypophosphatemia in premature infants are still debated. Indeed, inadequate supply of PO4 in the first days of life represent a relevant issue [2, 4], particularly in countries like Italy, where the main PO4 source for PN (D-Fructose-1,6-diphosphate) is no longer available.

We report on a SGA ELBW neonate experiencing severe hypercalcemia due to hypophosphatemia with the aim of discussing pathogenesis, treatment and prevention of this rare condition.

## Case presentation

A female baby was born at 29 weeks’ gestation by coesarian section because of placental vascular dysfunction and severe intrauterine growth restriction (IUGR), weighing 600 g (SGA). Her mother was receiving 2000 IU of vitamin D3 and 450 mg of magnesium per day and had no electrolytes abnormalities. Family history was negative for calcium (Ca) metabolism disorders. She developed respiratory distress necessitating mechanical ventilation for 2 days, followed by non-invasive ventilation. Spontaneous closure of ductus arteriosus was observed. C-reactive protein was raised at 12 h of life, but normalized 3 days after starting antibiotics. Blood culture was negative. She received PO4-free total PN for 2 days, containing amino acids up to 2.5 g/kg/day and Ca 0.5 mmol/kg/day. On day 3 of life, minimal enteral feeding was started using boluses of plain expressed breast milk (EBM), with gradual increase in the volume. Over the first 5 days of life, serum Ca concentrations were normal (between 7.8 mg/dl and 8.5 mg/dl).

**Fig. 1:**
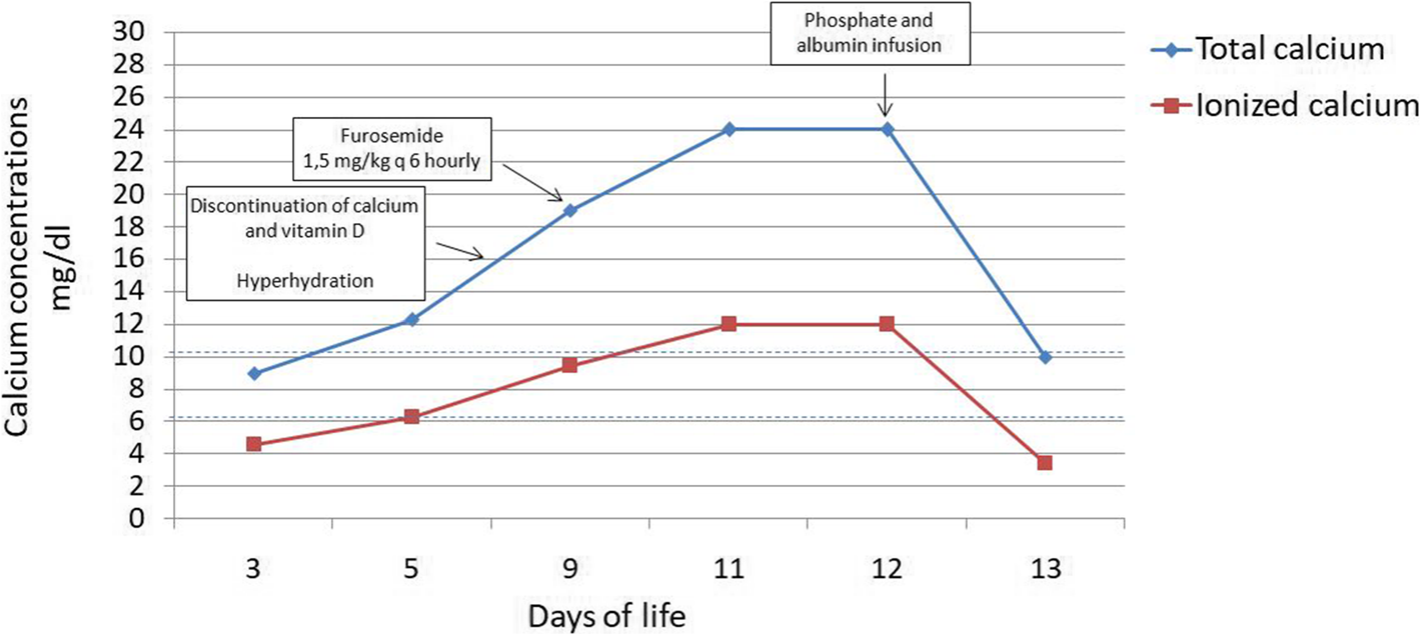
Onset and progress of hypercalcemia. Dotted lines indicate the normal range of serum corrected total calcium concentration for infants

Since day 6, she became hypercalcemic and Ca concentrations rose progressively (Fig. 1). Despite decrease and eventual discontinuation of parenteral Ca and Vitamin D delivery, corrected Ca concentrations reached a peak of 24 mg/dl. Diagnostic work-up revealed PO4 1.4 mg/dl (nv 4.4–8), magnesium 2.2 mg/dl (nv 1.7–2.5), sodium 137 mmol/L, potassium 3.8 mmol/L, albumin 1.8 g/dl (nv 3.3–4.5), alkaline phosphatase (ALP) 377 IU/L (nv 77–375), creatinine 1.06 mg/dl, urea 0.75 g/l, urine Ca/creatinine ratio 2.8 mmol/mmol (nv 0.09–2.2), urine PO4/creatinine ratio 1 mmol/mmol (nv 1.2–19), calcitonin 27 pg/ml (nv 0.8–9.9), normal thyroid function and suppressed parathyroid hormone (PTH) 4.8 pg/ml (nv 7.5–53.5), suggesting PTH-independent hypercalcemia. The infant was asymptomatic, except for mild polyuria.

Hypercalcemia did not improve after hyperhydration (220 ml/kg/day) and IV furosemide (1.5 mg/kg every 6 h) (Fig. 1). Given the rather small amount of EBM (16 ml/day), enteral nutrition was continued. Introduction of sodium glycerophosphate in the PN solution (PO4 80 mg/kg/day) resulted in a prompt decrease in Ca concentrations to 10 mg/dl within 24 h, with transient symptoms of hypocalcaemia (ionized Ca 3.4 mg/dl), which could be in part favored by contemporary infusion of albumin (Fig. 1). Therefore, the rate of infusion of sodium glycerophosphate was initially lowered and then a balanced infusion of Ca and PO4 was kept, in order to maintain normal Ca and PO4 concentrations. Four days after the introduction of PO4, PTH normalized (41.5 pg/ml, nv 7.5–53.5), while ALP was slightly raised at 788 IU/L, likely due to transient hypocalcemia. Within 3 weeks, her bone profile fully normalized (ALP 473 IU/L; PTH 39 pg/ml) and, due to good tolerance to formula milk feeds, Ca and PO4 supplements were discontinued. The baby had no evidence of renal or brain calcifications on repeated ultrasound scans nor bone lesions on x-ray.

## Discussion and conclusions

We report on a SGA ELBW neonate experiencing sustained severe hypercalcemia since the first week of life, associated with hypophosphatemia while on PN. This case provides insights on the mechanisms of Ca dysregulation in hypophosphatemic infants, emphasizing appropriate investigations, prevention and treatment aspects of this rare condition.

Severe hypercalcemia, defined as total serum Ca > 14 mg/dL [6], is uncommon in newborns. Although often asymptomatic, it may have important sequelae, such as brain calcifications and nephrocalcinosis possibly leading to distal tubular dysfunction, urinary stones, and renal failure [5]. Therefore, this condition requires prompt investigations and therapeutic interventions.

Neonatal hypercalcemia recognizes mainly PTH-independent causes [7, 8], such as sepsis [7], iatrogenic (drugs, vitamin A intoxication or excess Ca intake), maternal [9] or neonatal Vitamin D excess (including subcutaneous fat necrosis, hypophosphatemia, excess intake, and granulomatous diseases), congenital syndromes (eg hypocalciuric hypercalcemia, hypophosphatasia, blue diaper syndrome, Williams syndrome, Jansen metaphyseal chondrodysplasia), severe dysthyroidism or idiopathic [8].

In our case, the late onset and the persistence of severe hypercalcemia despite no administration of Ca and Vitamin D, along with low PO4 concentrations indicate hypophosphatemia as a key pathogenic factor. The mechanisms by which hypophosphatemia can cause hypercalcemia are not completely understood. Inhibition of the secretion of FGF23, leading to increased activity of 1-alfa hydroxylase with production of 1,25(OH)2D [8] or alternatively excess release from bones of Ca along with PO4, aiming to compensate the hypophosphatemic state [10] have been proposed.

Data from our patient indirectly support the first theory. In fact, although Vitamin D metabolites were not measured, the infant initially exhibited biochemical signs of hypervitaminosis D, such as low renal phosphate excretion and ALP concentrations not elevated in spite of hypophosphatemia. Moreover, the prompt reduction in calcium concentrations, along with increase in PTH concentrations up to normal values following phosphate infusion may possibly result from reduced production of active vitamin-D metabolites.

Hypophosphatemic hypercalcemia is rare and may occur more frequently in preterm compared to infants at term [8], especially if IUGR/SGA [3, 11, 12], due to a complex combination of factors peculiar to this category of patients. During the third trimester of pregnancy babies exhibit the fastest bone mineralization with great requirements of Ca and PO4 and thus preterm infants have depleted stores of these electrolytes [13]. Moreover, hypophosphatemia in ELBW may be worsen by a sort of re-feeding syndrome [13], also known as Placental Incompletely Restored Feeding syndrome, occurring after introduction of early high-energy PN in babies experiencing intrauterine nutritional deprivation and characterized by intracellular redistribution and increased reprocessing of electrolytes stimulated by insulin [2, 3, 14, 15]. Finally, hypophosphatemia in neonates can be exacerbated by renal losses, sepsis [4] and use of breast milk (which contains relatively large content of Ca compared to PO4) [16]. Although as mentioned above sepsis may also cause hypercalcemia, likely via production of 1,25(OH)2D by extrarenal macrophages [17] or interleukine-induced bone resorption [18], our case suggests poor relevance of this mechanism, as Ca concentrations continued to rise despite the resolution of early-onset sepsis.

Based on these considerations, monitoring and nutritional strategies for prevention of hypophosphatemia appear of paramount importance.

Established monitoring protocols are currently lacking. It has been suggested that, evaluation of PO4 concentration and other biochemical features of re-feeding syndrome should be performed by the third day of life in infants at risk, including VLBW or ELBW neonates receiving PN and repeated every 2 or 3 days [4, 13] or even twice daily before stabilization [19], by using reference ranges appropriate to preterm neonates [20].

Although PO4 supplementation is recommended from the third day of life [19], it has been shown that early introduction (even from the first day of life) in ELBW infants is safe [21–23] and results in lower incidence of Ca abnormalities and severe hypercalcemia, as well as in improved Ca retention [16, 24–26]. In addition, it might protect from negative effects exerted by hypophosphatemia on energy balance of several organs [4]. Nevertheless, there is a discrepancy between current recommendations on parenteral mineral supplementation. In fact, while guidelines by the American Academy of Pediatrics recommend PN supply of Ca and PO4 of 1.5–2 mmol/kg/day with Ca:PO4 ratio 1.1–1.3:1 [27], the European Society of Pediatric Gastroenterology, Hepatology, and Nutrition recommend 0.8–2 mmol/kg/day of Ca, and 1–2 mmol/kg/day of PO4, with a Ca: PO4 ratio of 0.8–1:1 [19]. In this respect, several studies [2, 25, 26] indicate that equimolar Ca:PO4 ratio might be more appropriate to meet the great PO4 requirement in the early post-natal period in VLBW infants. Indeed, in the study by Christman et al. [26] hypophosphatemia was detected in up to 34% of babies, despite providing the maximum recommended doses of PO4 for preterm infants with a Ca:PO4 ratio of 1.56. Recently, concern has been raised in countries like Italy where D-Fructose-1,6-diphosphate, the most common PO4 source for PN, is no longer available, and its alternative sodium glycerophosphate has to be imported from abroad. Furthermore, a relationship between amino acid intake and the risk of hypophosphatemia has been noted in preterm infants [2]. Although this suggests that PO4 requirement calculation should also take into account amino acid supply, current evidence is limited to provide clear recommendations in these patients [28].

As far as it concerns treatment, consistent with previous reports [29, 30], our case highlights that hypophosphatemic hypercalcemia does not benefit from treatments commonly used for PTH-indipendent hypercalcemia (such as hyperhydration, furosemide, subcutaneous calcitonin, steroids), but improves dramatically after iv or even oral [29] administration of PO4. Although in theory a drop in ionized calcium may be observed during correction of hypophosphatemia, so far this has never been reported in neonatal hypophosphatemic hypercalcemia. Therefore, given that we used common therapeutic doses of PO4, we hypothesize that contemporary infusion of albumin might have favored symptomatic hypocalcemia. Finally, our case highlights the need for regular check of Ca and PO4 concentrations, even every 2 hours [4], during PO4 infusion.

In conclusion, clinicians should be aware of the possible occurrence of life-threatening severe hypercalcemia in infants with or at risk to develop hypophosphatemia. Our case provides additional information regarding the mechanisms of Ca dysregulation in hypophosphatemic infants and confirms that hypophosphatemic hypercalcemia can only be managed with infusion of PO4. Current strategies for monitoring of phosphatemia and for adequate PO4 supplementation of in the first weeks of life in premature infants need to be implemented.

## Data Availability

Data sharing is not applicable to this article as no datasets were generated or analysed during the current study.

## Abbreviations

PN: Parenteral nutrition
PO4: Phosphate
Ca: Calcium
IUGR: Intrauterine growth retardation
PTH: Parathyroid hormone
ALP: Alkaline phosphatase
ELBW: Extremely low birth weight
VLBW: Very low birth weight
SGA: Small for gestational age
EBM: Expressed breast milk
